# Comparative Effects of Flurbiprofen and Benzydamine on COX-2/PGE_2_ and Inflammatory Cytokine Release in Polyinosinic:polycytidylic Acid-Stimulated Human Tonsil and Bronchial Epithelial Cells

**DOI:** 10.3390/ijms27177705

**Published:** 2026-08-28

**Authors:** Emmanuel Mfotie Njoya, Maria Spears, Thomas Hallett, Philippa Peters, Fiona Burke, Olumayokun A. Olajide

**Affiliations:** 1 Department of Pharmacy, School of Applied Sciences, University of Huddersfield, Huddersfield HD1 3DH, UK; e.mfotienjoya@hud.ac.uk; 2 Reckitt Benckiser Health Limited, Dansom Lane, Hull HU8 7DS, UK; maria.spears@reckitt.com (M.S.); thomas.hallett@reckitt.com (T.H.); philippa.peters@reckitt.com (P.P.); fiona.burke@reckitt.com (F.B.)

**Keywords:** flurbiprofen, benzydamine, pharyngitis, PGE_2_, COX-2, cytokines, chemokines

## Abstract

Topical non-steroidal anti-inflammatory drugs (NSAIDs) such as flurbiprofen and benzydamine may be used for the symptomatic relief of pharyngitis, triggered by infections. The onset and spectrum of anti-inflammatory activity of both drugs were compared in human tonsil (HTEpiC) and bronchial epithelial (BEAS-2B) cells stimulated with poly I:C against key inflammatory mediators. Cells were stimulated with poly I:C for 24 h and treated with flurbiprofen or benzydamine for 20 s, 2 min and 5 min. Production of prostaglandin E2 (PGE_2_), tumour necrosis factor-alpha (TNF-α), interleukin-6 (IL-6), interleukin-1 beta (IL-1β), interleukin-8 (IL-8), interleukin-18 (IL-18), monocyte chemotactic protein-3 (MCP-3) and C-X-C motif chemokine ligand 10 (CXCL10), as well as cyclooxygenase-2 (COX-2) protein expression, was evaluated. Flurbiprofen significantly (*p* < 0.05) reduced PGE_2_ production and COX-2 expression in both cell types within 20 s of treatment and maintained these effects at 2 and 5 min. However, benzydamine produced delayed, inconsistent inhibition. Although both drugs reduced poly I:C induced IL-6, IL-1β, IL-8 and IL-18 production, the magnitude and onset were greater with flurbiprofen. Flurbiprofen also reduced MCP-3 and CXCL10 in both cell types, alongside significant inhibition of caspase-1 activity. Benzydamine showed limited or no effect and less inhibition of caspase-1. With rapid suppression of the COX-2/PGE_2_ pathway, rapid onset and a broad inhibition spectrum, flurbiprofen more comprehensively modulates inflammatory processes in epithelial respiratory models.

## 1. Introduction

Pharyngitis is one of the most common reasons for seeking medical care in primary care settings [1,2]. In addition to bacterial and fungal infections, pharyngitis can also be caused by viruses [3,4], with rhinovirus and influenza being responsible for about 85% of cases [5]. Typically, viral infections activate several pro-inflammatory processes, resulting in the release of prostaglandins, cytokines and bradykinin [6,7].

Acute pharyngitis is usually self-limiting and rarely leads to serious outcomes in otherwise healthy individuals [8]. However, the condition is distressing due to most patients experiencing moderate to severe pain [9,10]. Regardless of the cause, pharyngeal inflammation is a consequence of injury to the epithelium, pro-inflammatory cytokine release, and recruitment of immune cells [11,12].

The inflammatory symptoms of pharyngitis are driven by the co-ordinated action of several mediators; prostaglandin E2 (PGE_2_) generated through cyclooxygenase-2 (COX-2) is particularly important because PGE_2_ has a central role in pain and inflammation. It acts in concert with mediators such as bradykinin, producing sore throat symptoms including swelling, pain, redness, heat and loss of function [13,14].

Furthermore, tissue production of pro-inflammatory cytokines, like tumour necrosis factor-alpha (TNF-α) and interleukins-1 (IL-1) and -6 (IL-6), has been reported [15,16] and can contribute to the activation of inflammatory cells within affected tissue. Consequently, suppression of COX-2/PGE_2_ signalling, together with modulation of the broader cytokine response, represents an important therapeutic strategy for symptom relief in acute pharyngitis.

Management of pharyngitis typically consists of symptomatic treatment aiming for pain relief [17]. Topical non-steroidal anti-inflammatory drugs (NSAIDs) remain a mainstay of treatment because of their ability to reduce inflammation-associated pain [18,19,20] and can be administered locally to the site of inflammation as lozenges or sprays to facilitate local bioavailability [21,22].

Investigation into the effects of pharmacological agents on pharyngitis have employed tonsil epithelial cells as the main in vitro model. Using UT-SCC-60A and UT-SCC-60B cell lines, Lange et al. (2009) demonstrated that tonsillar epithelial cells showed strong expression of TLR3 mRNA by responding significantly to stimulation with the synthetic TLR3 ligand, poly I:C, and secreting IL-6 and CXCL10/IP-10 [23]. Similarly, experiments using poly I:C to stimulate human primary tonsil epithelial cells resulted in elevated production of prostaglandin E2 (PGE_2_), tumour necrosis factor-alpha (TNFα) and interleukin-6 (IL-6) [24]. Studies by Lange et al. further demonstrated that tonsillar epithelial cells could recognise pathogen-associated molecular patterns (PAMPs) in *Streptococcus pyogenes*, such as lipoteichoic acid (LTA) and peptidoglycan (PGN) [23]. When used to stimulate human primary tonsil epithelial cells, PGN and LTA significantly induced increased production of pro-inflammatory mediators IL-6, IL-8, CXCL5, CXCL6, PGE_2_ and COX-2 [25].

**Figure 1:**
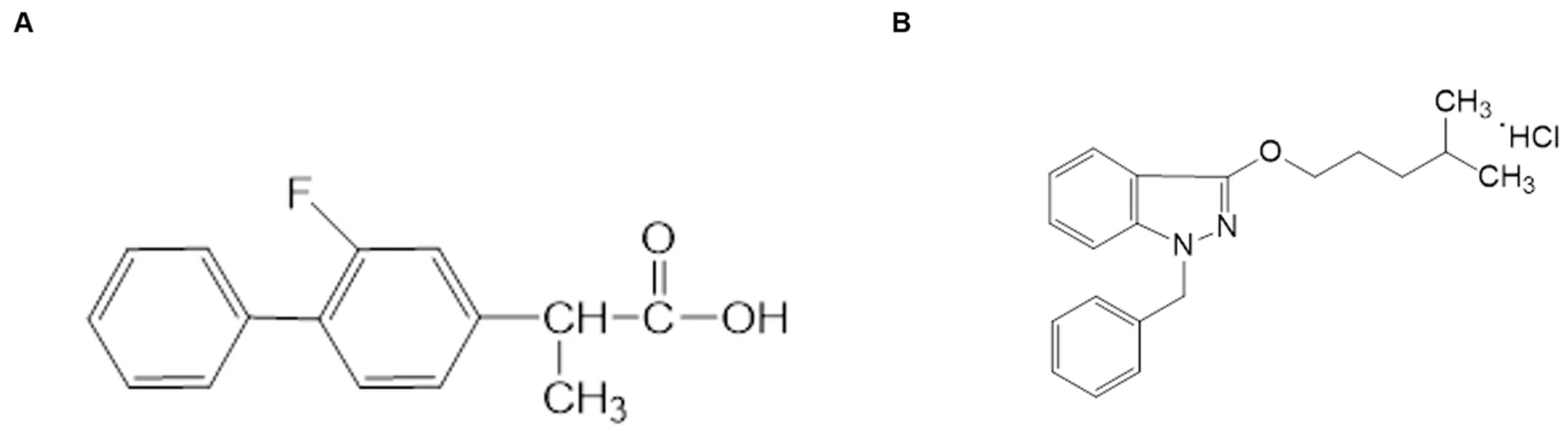
Chemical structures of flurbiprofen (**A**) and benzydamine hydrochloride (**B**).

Flurbiprofen, 2-(2-fluoro-4-biphenylyl) propionic acid (Figure 1A), is a propionic acid derivative cyclooxygenase-2 inhibitor used in the treatment of pain or inflammation in humans [26]. Flurbiprofen is used orally and topically for the symptomatic treatment of migraine and chronic inflammatory diseases, such as rheumatoid arthritis and osteoarthritis, to reduce pain and fever [27]. Flurbiprofen is formulated as a lozenge or spray for the symptomatic relief of pharyngitis, to act locally at lower concentrations than systemic administration, due to its suppressive effect on PGE_2_ release [28]. Despite its widely reported anti-inflammatory and analgesic activities, there is limited published data on its effects on pro-inflammatory mediators in models of pharyngitis.

Benzydamine (1-benzyl-3-[3-(dimethylamino)propoxy]-1H-indazole) (Figure 1B) is an NSAID that is used for topical treatment of multiple oral inflammatory conditions [29]. It is a weak inhibitor of cyclooxygenase, which reduces the production of PGE_2_ and other prostanoids at high concentrations [30]. Rather, the anti-inflammatory activity of this drug has been suggested to be linked to its ability to reduce the release of pro-inflammatory cytokines, such as TNF-α and IL-1β [31]. Benzydamine hydrochloride is formulated as topical preparations (lozenge and spray) for the symptomatic relief of pain in acute sore throat [32].

Therefore, the aim of this study was to compare the onset and breadth of anti-inflammatory activity of flurbiprofen and benzydamine in human tonsil epithelial cells stimulated with polyinosinic:polycytidylic acid (poly I:C), including modulation of the COX-2/PGE_2_, alongside evaluation of cytokine and chemokine (MCP-3 and CXCL10)-associated inflammatory responses. Investigations were also carried out using BEAS-2B bronchial epithelial cells, which were employed as a second cellular model of inflammation of the respiratory tract. To our knowledge, this is the first study demonstrating inhibitory effects of flurbiprofen on MCP-3 and CXCL10 production in respiratory epithelial cells following poly I:C stimulation.

## 2. Results

### 2.1. Flurbiprofen Reduced PGE_2_ Production More Rapidly than Benzydamine

Given the established role of COX-2/PGE_2_ production in inflammation-associated pain, the effects of flurbiprofen and benzydamine on PGE_2_ production were evaluated against control.

**Figure 2:**
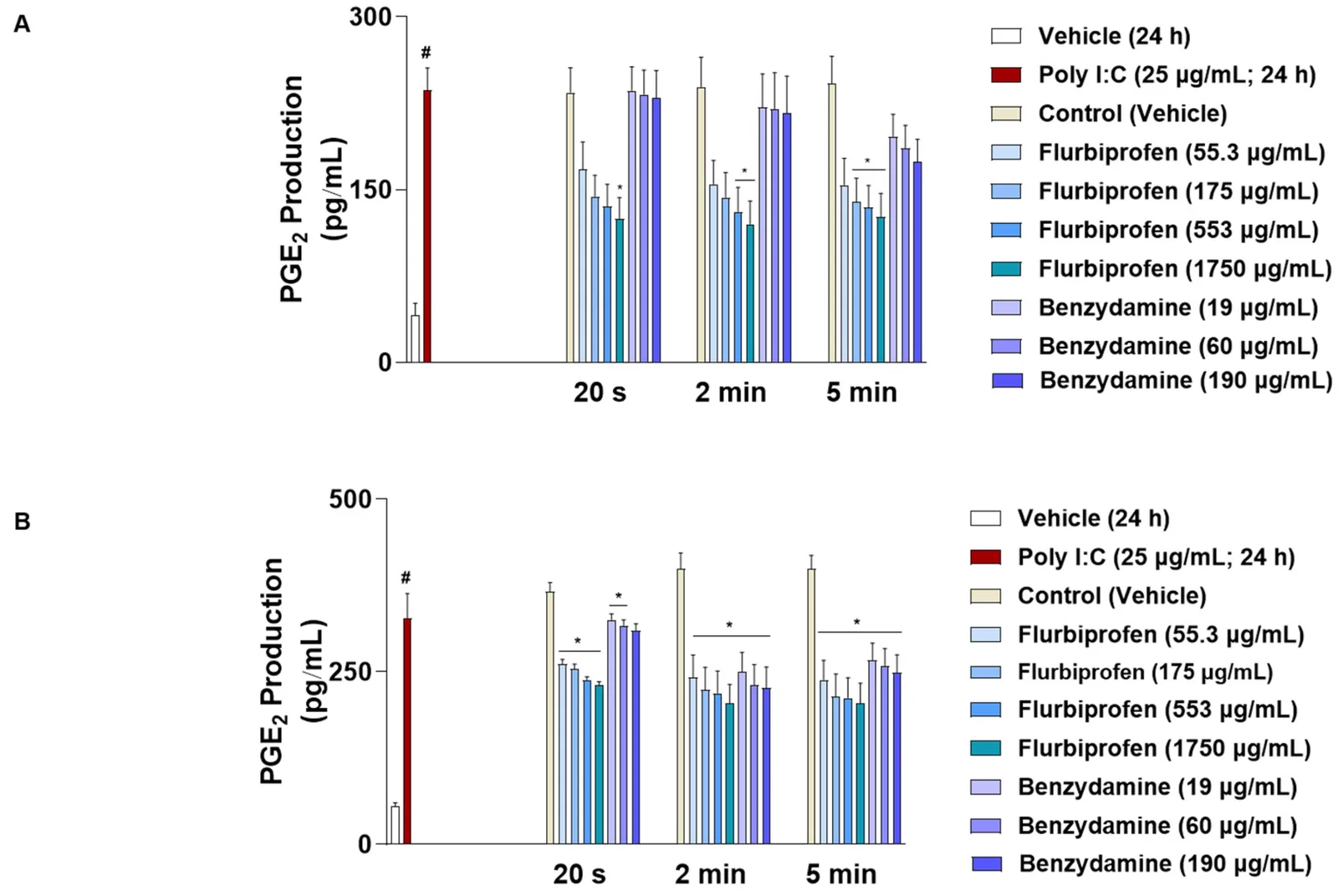
Onset of flurbiprofen (55.3, 175, 553 and 1750 µg/mL) and benzydamine (19, 60 and 190 µg/mL) action on the production of PGE_2_ in HTEpiC (**A**) and BEAS-2B cells (**B**) stimulated with poly I:C. Cells were stimulated with poly I:C (25 µg/mL) for 24 h, followed by drug treatment for 20 s, 2 min and 5 min. Levels of PGE_2_ were analysed in culture supernatants. Results are mean ± SEM for at least 3 independent experiments; # *p* < 0.05 in comparison to unstimulated cells, * *p* < 0.05 in comparison to untreated cells; one-way analysis of variance (ANOVA) followed by Tukey’s post hoc test.

Analyses of supernatants from poly I:C-stimulated HTEpiC cells revealed that treatment with flurbiprofen significantly (*p* < 0.05) reduced PGE_2_ production at 1750 µg/mL within 20 s of treatment and maintained this effect at 2 (553 and 1750 µg/mL) and 5 min (175, 553 and 1750 µg/mL) (Figure 2A).

Treatment with benzydamine did not result in a significant (*p* < 0.05) reduction in PGE_2_ until at all the time points (Figure 2A). Similar findings were observed in human BEAS-2B cells, where flurbiprofen and benzydamine significantly reduced PGE_2_ at all time points (Figure 2B).

### 2.2. Flurbiprofen Reduced COX-2 Protein to a Greater Degree and More Rapidly than Benzydamine

**Figure 3:**
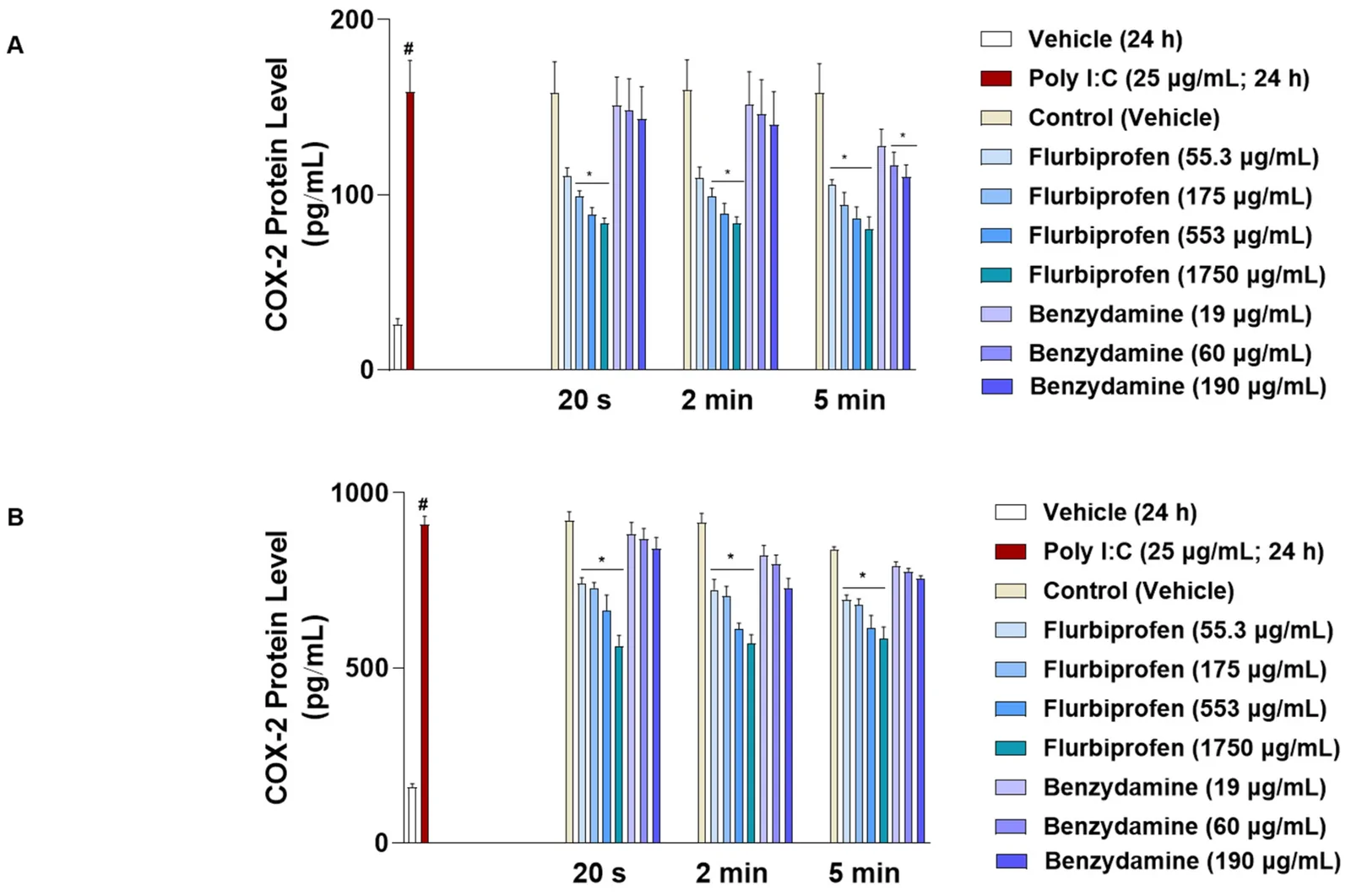
Onset of flurbiprofen (55.3, 175, 553 and 1750 µg/mL) and benzydamine (19, 60 and 190 µg/mL) action on protein levels of COX-2 in HTEpiC (**A**) and BEAS-2B cells (**B**) stimulated with poly I:C. Cells were stimulated with poly I:C (25 µg/mL) for 24 h, followed by drug treatment for 20 s, 2 min and 5 min. Levels of COX-2 protein were analysed in cell lysates. Results are mean ± SEM for at least 3 independent experiments; # *p* < 0.05 in comparison to unstimulated cells, * *p* < 0.05 in comparison to untreated cells; one-way analysis of variance (ANOVA) followed by Tukey’s post hoc test.

Consistent with the effects observed on PGE_2_ production, flurbiprofen significantly (*p* < 0.05) and concentration-dependently reduced COX-2 protein levels in poly I:C-stimulated HTEpiC cells at concentrations of 175, 553 and 1750 µg/mL at 20 s, 2 min and 5 min (Figure 3A). In comparison, reductions in COX-2 expression following benzydamine treatment were not evident until 5 min post-treatment (at higher concentrations, e.g., 60, 190 µg/mL).

In BEAS-2B cells, flurbiprofen similarly produced significant reductions in COX-2 expression at all concentrations and assessed time points (20 s, 2 min and 5 min; Figure 3B). Under the same experimental conditions, benzydamine did not significantly reduce COX-2 protein expression at any time point examined (Figure 3B).

### 2.3. Effects of Flurbiprofen and Benzydamine on the Production of Pro-Inflammatory Cytokines and Chemokines in Poly I:C-Stimulated HTEpiC Cells

Following evaluation of COX-2/PGE_2_ production, the effects of flurbiprofen and benzydamine on poly I:C-induced cytokine and chemokine production were investigated in HTEpiC cells. Both drugs reduced the production of key pro-inflammatory cytokines, although differences in onset and magnitude of response were observed across individual mediators.

**Figure 4:**
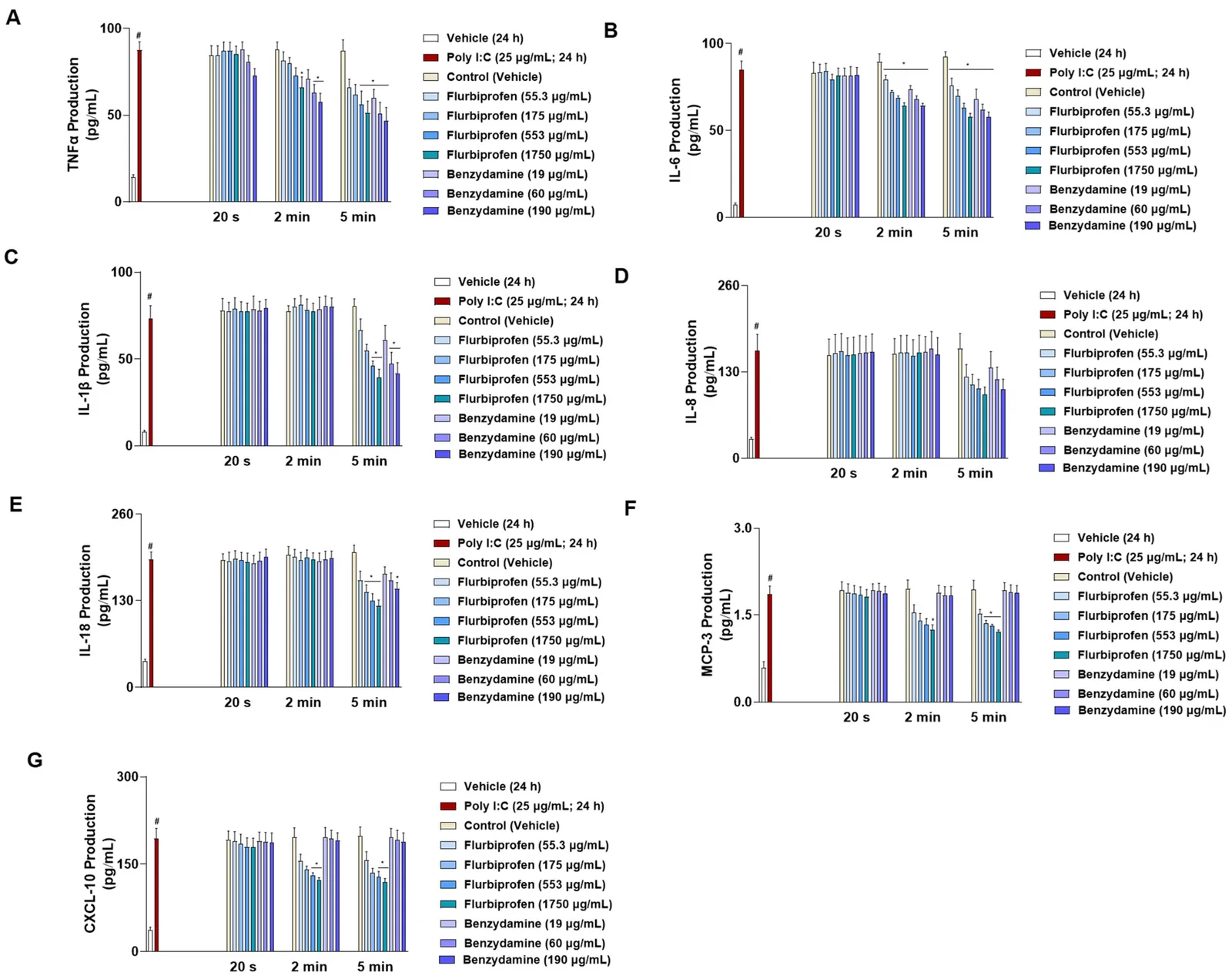
Comparative reduction in the production of TNF-α (**A**), IL-6 (**B**), IL-1β (**C**), IL-8 (**D**), IL-18 (**E**), MCP-3 (**F**) and CXCL10 (**G**) by flurbiprofen (55.3, 175, 553 and 1750 µg/mL) and benzydamine (19, 60 and 190 µg/mL) in HTEpiC cells stimulated with poly I:C. Cells were stimulated with poly I:C (25 µg/mL) for 24 h, followed by drug treatment for 20 s, 2 min and 5 min. Levels of pro-inflammatory mediators were analysed in culture supernatants using ELISA. Results are mean ± SEM for at least 3 independent experiments; # *p* < 0.05 in comparison to unstimulated cells, * *p* < 0.05 in comparison to untreated cells; one-way analysis of variance (ANOVA) followed by Tukey’s post hoc test.

For TNF-α, neither flurbiprofen nor benzydamine reduced cytokine production at 20 s post-treatment (Figure 4A). By 2 min, both drugs reduced TNF-α levels, and significant inhibition was evident across most treatment conditions after 5 min. Similar patterns were observed for IL-6, with both drugs demonstrating significant reductions from 2 min onwards (Figure 4B).

Reductions in IL-1β and IL-18 were not evident until 5 min post-treatment (Figure 4C–E). At this time point, both flurbiprofen and benzydamine significantly reduced these mediators in a concentration-dependent manner. Although both treatments were active, reduction in IL-18 were generally greater in cells treated with flurbiprofen. Both drugs did not produce a significant (*p* < 0.05) reduction in IL-8.

More distinct differences between treatments were observed for chemokine production. While neither treatment reduced MCP-3 or CXCL10 after 20 s, flurbiprofen significantly and concentration-dependently reduced both mediators at 2 and 5 min post-treatment (Figure 4F,G). However, benzydamine did not significantly reduce MCP-3 or CXCL10 at any of the time points examined.

Collectively, these findings indicate that both flurbiprofen and benzydamine modulate poly I:C-induced cytokine responses in HTEpiC cells, whereas differences between the treatments are more apparent in the regulation of chemokine production.

### 2.4. Comparative Effects of Flurbiprofen and Benzydamine in Poly I:C-Stimulated BEAS-2B Cells

**Figure 5:**
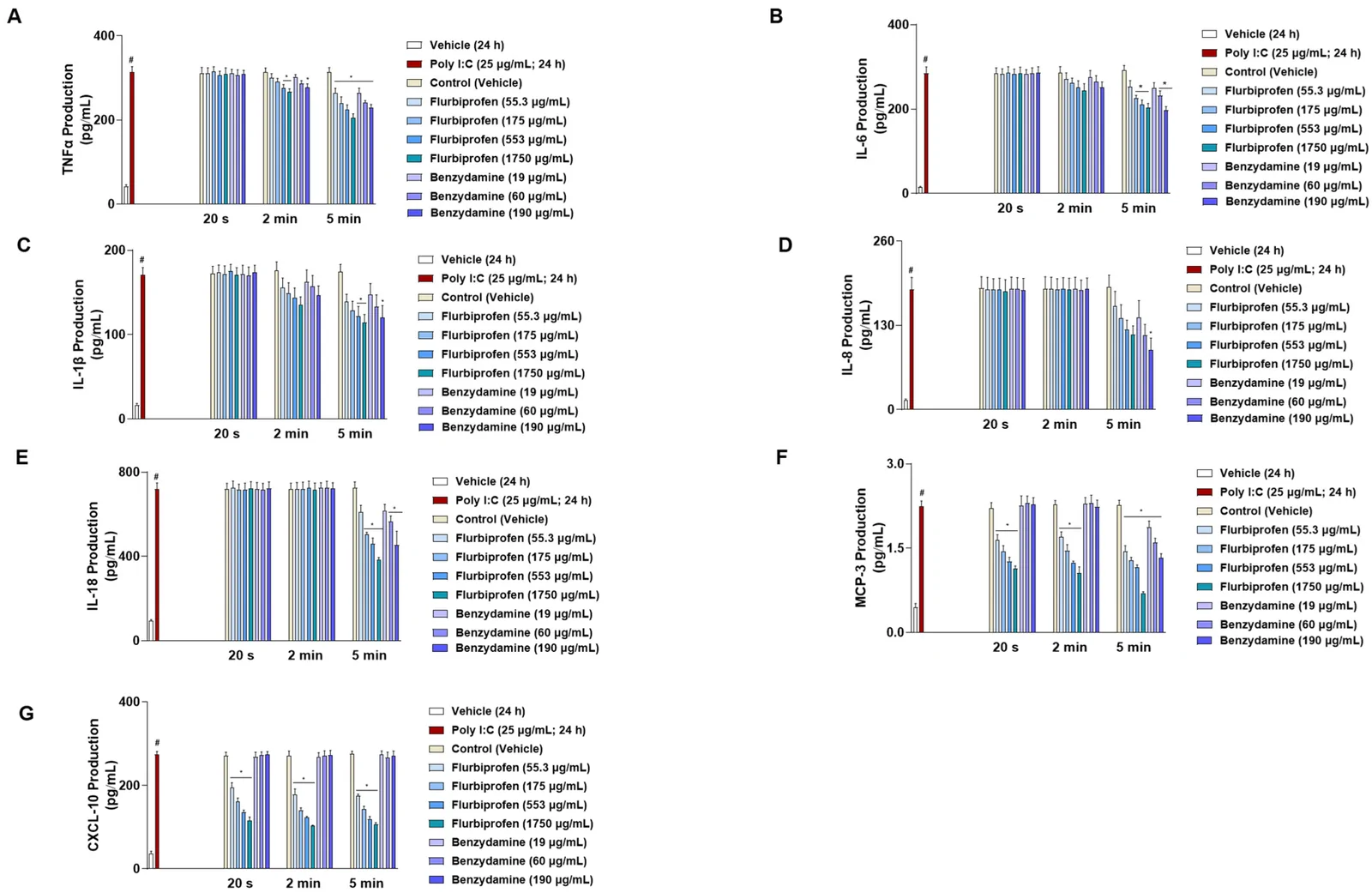
Comparative reduction in the production of TNF-α (**A**), IL-6 (**B**), IL-1β (**C**), IL-8 (**D**), IL-18 (**E**), MCP-3 (**F**) and CXCL10 (**G**) by flurbiprofen (55.3, 175, 553 and 1750 µg/mL) and benzydamine (19, 60 and 190 µg/mL) in BEAS-2B cells stimulated with poly I:C. Cells were stimulated with poly I:C (25 µg/mL) for 24 h, followed by drug treatment for 20 s, 2 min and 5 min. Levels of pro-inflammatory mediators were analysed in culture supernatants using ELISA. Results are mean ± SEM for at least 3 independent experiments; # *p* < 0.05 in comparison to unstimulated cells, * *p* < 0.05 in comparison to untreated cells; one-way analysis of variance (ANOVA) followed by Tukey’s post hoc test.

Similar patterns were observed in BEAS-2B cells. Both flurbiprofen and benzydamine significantly reduced TNF-α and IL-6 production after 5 min of treatment (Figure 5A,B). While both drugs demonstrated activity against these cytokines, reductions in TNF-α were more pronounced with flurbiprofen.

Poly I:C-induced IL-1β production was significantly (*p* < 0.05) reduced by both treatments at 5 min, with flurbiprofen producing greater inhibition than benzydamine (Figure 5C). A significant reduction in IL-8 was observed with benzydamine (190 µg/mL) at 5 min, while reduction by flurbiprofen was not significant (*p* < 0.05) (Figure 5D). Flurbiprofen (175, 553 and 1750 µg/mL) and benzydamine (60 and 190 µg/mL) significantly (*p* < 0.05) reduced IL-18, with flurbiprofen producing more pronounced suppression of IL-18 (Figure 5E).

Differences between treatments were most apparent for chemokine regulation. Flurbiprofen significantly reduced MCP-3 levels at all time points evaluated, whereas benzydamine showed activity only at 5 min and to a lesser extent (Figure 5F). Similarly, flurbiprofen significantly suppressed CXCL10 production at all time points, whereas benzydamine did not significantly affect CXCL10 levels under any condition tested (Figure 5G).

### 2.5. Flurbiprofen Reduced Caspase-1 Activity in HTEpiC and BEAS-2B Cells

**Figure 6:**
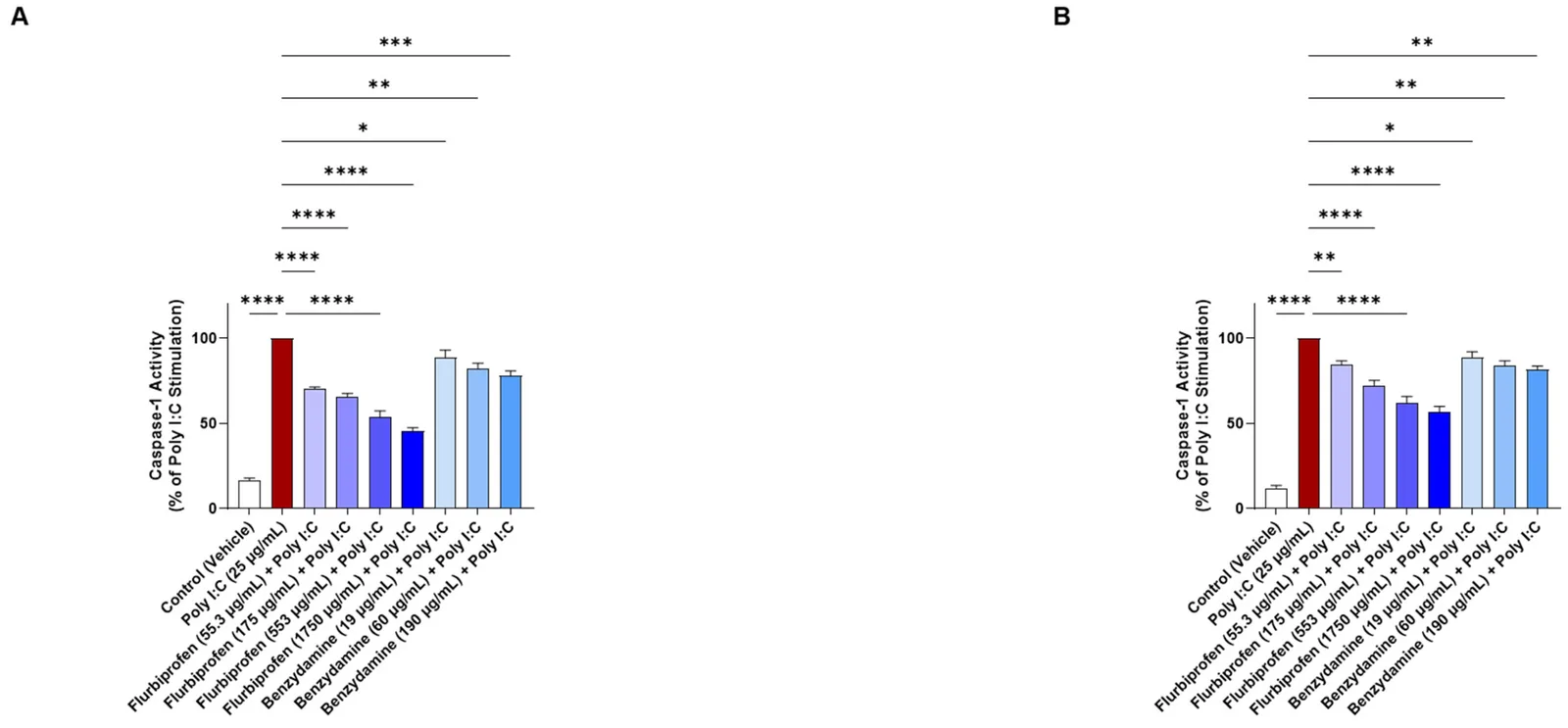
Effects of flurbiprofen (55.3, 175, 553 and 1750 µg/mL) and benzydamine (19, 60 and 190 µg/mL) on caspase-1 activity in HTEpiC (**A**) and BEAS-2B cells (**B**) stimulated with poly I:C (25 µg/mL) for 24 h. Caspase-1 activity was measured using Caspase-Glo^®^ inflammasome assay. Results are mean ± SEM for at least 3 independent experiments; * *p* < 0.05, ** *p* < 0.01, *** *p* < 0.001, **** *p* < 0.0001 in comparison to untreated cells; one-way analysis of variance (ANOVA) followed by Tukey’s post hoc test.

Because IL-1β and IL-18 production are dependent on caspase-1 activity, the effects of both drugs on inflammasome-associated signalling were investigated. In both HTEpiC (Figure 6A) and BEAS-2B cells (Figure 6B), flurbiprofen produced marked and concentration-dependent reductions in caspase-1 activity. Benzydamine demonstrated comparatively weaker effects in both cell types. These findings are consistent with the stronger inhibition of IL-1β and IL-18 observed following flurbiprofen treatment.

## 3. Discussion

Regardless of the aetiology of pharyngitis, inflammation has been shown to be the major contributor to the pathogenesis of this condition [33,34], including sore throat pain, erythema and oedema.

In viral pharyngitis, respiratory viruses trigger host immune responses involving significant release of pro-inflammatory mediators, including prostaglandins, cytokines and chemokines. Among these mediators, COX-2-derived prostaglandin E2 (PGE_2_) is particularly relevant because of its established role in amplifying inflammation and sensitising sensory nerve endings associated with pain perception [11]. Consequently, rapid suppression of inflammatory signalling pathways, particularly the COX-2/PGE_2_ axis, may be expected to contribute to symptomatic benefit in acute pharyngitis.

In the present study, poly I:C stimulation induced marked increases in PGE_2_, COX-2, TNF-α, IL-6, IL-1β, IL-8, IL-18 MCP-3, CXCL10 and caspase-1 in both HTEpiC and BEAS-2B cells, potentially supporting the utility of these models for investigating host inflammatory responses relevant to viral pharyngitis. These findings are consistent with previous reports demonstrating increased cytokine production and activation of inflammatory pathways in epithelial cells following poly I:C stimulation [23,35,36].

Both flurbiprofen and benzydamine reduced inflammatory responses induced by poly I:C stimulation; however, differences were observed in the timing and extent of activity across specific pathways. The clearest distinction between the treatments was seen within COX-2/PGE_2_ production. Flurbiprofen significantly reduced both COX-2 expression and PGE_2_ production within 20 s in HTEpiC and BEAS-2B cells, whereas benzydamine produced more delayed effects that varied between cell types and endpoints. Given the established role of PGE_2_ in inflammatory pain signalling and symptom generation in sore throat, these findings suggest that modulation of this pathway may contribute to differences in the onset of anti-inflammatory activity observed between the treatments.

Previous studies have demonstrated inhibition of virus-induced PGE_2_ production by flurbiprofen in respiratory epithelial cells [28]. The present findings extend these observations by demonstrating rapid effects on both PGE_2_ production and COX-2 expression in two epithelial cell models relevant to the respiratory tract. Notably, benzydamine also reduced PGE_2_ production under the same experimental conditions despite its generally weak COX-2-inhibitory activity [30,37]. This observation is consistent with previous reports suggesting that benzydamine may influence prostaglandin synthesis through effects on cyclooxygenase and phospholipase A_2_ pathways [31,37].

Beyond COX-2/PGE_2_, both drugs demonstrated activity against multiple cytokines induced by poly I:C stimulation. In HTEpiC cells, both flurbiprofen and benzydamine reduced TNF-α and IL-6 production, with benzydamine showing an early effect on TNF-α at the highest concentration tested. Similarly, both treatments reduced IL-1β, IL-8 and IL-18, although these effects were generally observed at later time points.

While differences between treatments were less pronounced for TNF-α and IL-6, flurbiprofen tended to produce greater reductions in IL-1β and IL-18, particularly in BEAS-2B cells. These findings suggest that both NSAIDs can modulate cytokine responses associated with viral inflammation, although the magnitude and timing of effects vary according to the mediator examined.

More distinct differences between treatments emerged when chemokine responses were evaluated. Flurbiprofen significantly reduced MCP-3 and CXCL10 production in both epithelial models, whereas benzydamine showed limited activity against MCP-3 and no significant effect on CXCL10. As these chemokines contribute to immune cell recruitment and amplification of local inflammatory responses during viral infection, these findings suggest that flurbiprofen may exert effects across a broader range of inflammatory pathways than those directly associated with prostaglandin synthesis alone.

To our knowledge, this is the first study demonstrating inhibitory effects of flurbiprofen on MCP-3 and CXCL10 production in respiratory epithelial cells following poly I:C stimulation. These observations extend previous evidence of its anti-inflammatory activity beyond modulation of traditional cytokine responses [38,39].

Activation of the NLRP3 inflammasome is an important mechanism linking viral sensing to the production of IL-1β and IL-18 [40,41,42,43]. Because flurbiprofen produced greater reductions in these mediators than benzydamine, we explored whether inflammasome-associated signalling could contribute to these effects.

Flurbiprofen produced marked and concentration-dependent inhibition of caspase-1 activity in both epithelial cell models, whereas benzydamine demonstrated comparatively weak effects. Notably, the pattern of caspase-1 inhibition mirrored the effects observed for IL-1β and IL-18, suggesting that modulation of inflammasome-associated pathways may contribute to the broader anti-inflammatory profile observed with flurbiprofen. These findings are consistent with previous reports demonstrating inhibition of NLRP3 inflammasome signalling by flurbiprofen in other inflammatory models [36]. Nevertheless, direct evaluation of NLRP3 activation was beyond the scope of the present study, and further mechanistic investigations are warranted.

Collectively, the findings demonstrate that both flurbiprofen and benzydamine reduce inflammatory mediator release in an epithelial model of viral pharyngitis, as well as inflammation of the respiratory tract in general. However, the most notable differences between treatments were observed in the rapid modulation of COX-2/PGE_2_ production and in the spectrum of inflammatory mediators affected.

Flurbiprofen demonstrated earlier and greater suppression of COX-2 and PGE_2_ and greater reduction in selected mediators (IL-1β and IL-18), and reduced a wider range of downstream mediators, including MCP-3, CXCL10 and caspase-1 activity. These results suggest that, in addition to its established effects on prostaglandin production, flurbiprofen may influence multiple inflammatory pathways relevant to sore throat pathophysiology. Further studies are required to elucidate the molecular mechanisms underlying these observations and to determine their translational relevance to clinical outcomes in pharyngitis.

## 4. Materials and Methods

### 4.1. Materials

Flurbiprofen (Lot 8455; ≥98%) was obtained from Pharmaron (Beijing, China), while benzydamine hydrochloride (Lot R23L014; ≥98.2%) was purchased from ThermoFisher Scientific (Paisley, UK). Polyinosinic–polycytidylic acid potassium salt (100%) was purchased from Merck Life Science (Gillingham, UK).

### 4.2. Cell Culture

Human tonsil epithelial (HTEpiC) cells were purchased from Generon Ltd. (Slough, UK), cultured in tonsil epithelial cell medium (ScienCell, Carlsbad, CA, USA) on a poly-L-lysine (Merck, Gillingham, UK)-coated flask and incubated at 37 °C in a 5% CO_2_ humidified incubator. Cells were used for pharmacological studies between passages 3 and 14.

Human bronchial epithelial (BEAS-2B) cells were obtained from CLS Cell Lines Service GmbH, Eppelheim, Germany, and cultured as described previously [44]. Cells were employed in pharmacological assays between passages 4 and 16.

Viability assays using the MTS assay revealed that stimulation with poly I:C for 24 h, followed by treatment with flurbiprofen (175, 553 and 1750 µg/mL) or benzydamine (19, 60 and 190 µg/mL) for 20 s, 2 min and 5 min, did not affect cell viability.

### 4.3. Treatment and Stimulation

Flurbiprofen was dissolved in DMSO (≥99.7%) and tested at concentrations of 55.3, 175, 553 and 1750 µg/mL. The highest concentration of 1750 µg/mL was based on the outcome of cell viability assays and the recommended clinical dose of 8.75 mg used in sore throat treatment when diluted in 5 mL of saliva [45]. The concentrations of benzydamine used were 19, 60 and 190 µg/mL. The highest concentration (190 µg/mL) was shown to be non-cytotoxic and based on a clinical dose of 3 mg dissolved in 5 mL of saliva.

For pharmacological experiments, HTEpiC cells were seeded into poly-L-lysine-coated 48-well tissue culture plates at a density of 2 × 10^5^ cells/mL in 500 µL culture medium, while BEAS-2B cells were seeded at a density of 2 × 10^5^ cells/mL into 48-well tissue culture plates coated with fibronectin, bovine collagen type I and bovine serum albumin (BSA) dissolved in 500 µL LHC basal medium (ThermoFisher Scientific, Paisley, UK). Cells were used for in vitro assays at 70% confluence.

Cultured HTEpiC or BEAS-2B cells were stimulated with poly I:C (25 µg/mL). After 24 h, cells were treated with either flurbiprofen or benzydamine without medium change. Control cells were treated with an equal volume of DMSO (≤0.2%).

Culture supernatants and cell lysates were collected at 20 s, 2 min and 5 min post-treatment for analyses and frozen at −80 °C prior to analyses.

### 4.4. Determination of PGE_2_

Culture supernatants collected at 20 s, 2 min and 5 min post-treatment were analysed for PGE_2_ production using a PGE_2_ parameter assay kit (R and D Systems, Abingdon, UK), according to the manufacturer’s instructions.

### 4.5. Cyclooxygenase-2 ELISA

Cell lysates obtained at 20 s, 2 min and 5 min post-treatment were evaluated for COX-2 protein levels with the human/mouse total COX-2 DuoSet IC ELISA (R and D Systems), following the manufacturer’s instructions.

### 4.6. Determination of Cytokine and Chemokine Production

Cell supernatants were analysed for levels of the following cytokines and chemokines using human ELISA kits, following the manufacturers’ instructions: TNF-α (ThermoFisher Scientific, Paisley, UK), IL-6 (ThermoFisher Scientific), IL-1β (ThermoFisher Scientific), IL-8 (ThermoFisher Scientific), IL-18 (ThermoFisher Scientific), MCP-3 (ThermoFisher Scientific) and CXCL10 (R and D Systems).

### 4.7. Caspase-Glo^®^ 1 Inflammasome Assay

Caspase-1 activity was evaluated using the Caspase-Glo^®^ inflammasome assay (Promega, Southampton, UK), as described earlier [41]. Culture supernatants were mixed with Caspase-Glo^®^ 1 reagent in a 96-well plate. This was followed by luminescent measurement of caspase-1 activity using a multimodal microplate reader (Infinite™ M200 PRO; Tecan, Grödig, Austria).

### 4.8. Statistical Analysis

Data from all experiments are mean ± SEM for at least 3 independent experiments. In all experiments, effects of drug treatments were compared with untreated cells which were stimulated with poly I:C (control). Data were analysed using one-way analysis of variance (ANOVA) followed by Tukey’s post hoc test. Values with *p* < 0.05 were statistically significant. Statistical analyses were carried out with GraphPad Prism Software version 10.5.0.

## Data Availability

The original contributions presented in this study are included in the article. Further inquiries can be directed to the corresponding author.
